# Safety of acupuncture by Korean Medicine Doctors: a prospective, practice-based survey of 37,490 consultations

**DOI:** 10.1186/s12906-022-03782-z

**Published:** 2022-11-18

**Authors:** Jiyoon Won, Jun-Hwan Lee, Heejung Bang, Hyangsook Lee

**Affiliations:** 1 KM Science Research Division, Korea Institute of Oriental Medicine, Daejeon, Korea; 2 Korean Convergence Medical Science, KIOM School, University of Science & Technology (UST), Daejeon, Korea; 3 Division of Biostatistics, Department of Public Health Sciences, University of California, Davis, CA USA; 4 Acupuncture & Meridian Science Research Centre, College of Korean Medicine, Kyung Hee University, Seoul, Korea; 5 Department of Science in Korean Medicine, College of Korean Medicine, Graduate School, Kyung Hee University, Seoul, Korea

**Keywords:** Acupuncture, Adverse events, Causality, Frequency, Severity, Korean Medicine Doctor, Safety

## Abstract

**Background:**

To evaluate safety of acupuncture treatment by Korean Medicine Doctors (KMDs), a prospective, practice-based survey on adverse events (AEs) associated with acupuncture was conducted.

**Methods:**

From July 2016 to October 2017, KMDs were invited to participate in an online survey. Frequency was calculated as the number of AEs per 10,000 treatments; severity was assessed with the Common Terminology Criteria for Adverse Events Grading (Severity) Scale; and causality was evaluated using the World Health Organisation-Uppsala Monitoring Centre system for standardised case causality assessment. Associations between AE occurrence and KMDs’ type of practice/clinical experience and patient age/gender/current medication(s) were analysed.

**Results:**

Data on 37,490 acupuncture treatments were collected from 222 KMDs. At least one AE was reported from 4,518 acupuncture treatments, giving a frequency rate of 1,205 per 10,000 acupuncture treatments; this increased to 4,768 treatments when administrative problems related to defective devices or medical negligence were added, for a rate of 1,272 per 10,000 acupuncture treatments. Commonly reported AEs were bleeding, needle site pain, and bruising. Approximately 72.9% of AEs/administrative problems were assessed as they certainly occurred by acupuncture treatment in causality assessment. Most AEs/administrative problems were considered mild in severity and two life-threatening AEs were resolved with no sequelae. Compared to males, female patients were more likely to experience AEs and KMDs’ clinical experience was not associated with reported AE occurrence.

**Conclusions:**

Although acupuncture-associated AEs occur commonly, they are largely transient and mild. Acupuncture performed by qualified KMDs may serve as a reliable medical treatment with acceptable safety profiles.

**Supplementary Information:**

The online version contains supplementary material available at 10.1186/s12906-022-03782-z.

## Background

Acupuncture, as one of the most commonly used complementary and alternative medicine therapeutic modalities, is increasingly popular across the globe [1]. While evidence of the efficacy of acupuncture has accumulated over decades, mainly from studies of chronic pain [2], its safety has been less extensively investigated. Although previous large-scale studies from the UK [3], Germany [4], and Japan [5] reported an acceptable safety profile for acupuncture treatment, outcomes may differ according to the clinical and cultural context where it is performed.

In South Korea, acupuncture is among the most common treatment modalities in Korean Medicine practice, is legally practised only by Korean Medicine Doctors (KMDs) and is routinely reimbursed by the national health insurance system. Common acupuncture-related techniques include manual acupuncture, electroacupuncture, moxibustion, cupping, auricular acupuncture, and pharmacopuncture [6, 7]. Previous studies of acupuncture and pharmacopuncture reported that these treatments are a relatively safe option when performed by qualified KMDs [8, 9]. These studies were retrospectively conducted in one or two hospitals, such that they may not be entirely free from recall bias and may lack applicability to small private practices that account for >97% of Korean Medicine institutions [8–10]. While a prospective study involving 13 KMDs reported on 99 AEs from among 89 patients [11], its sample size was smaller compared with previous studies from the UK [3], Germany [4] or Australia [12], making it virtually impossible to detect rare AEs. However, rare but serious AEs do occur judging by reports from emergency departments of tertiary hospitals [13] and the Korea Medical Dispute Mediation and Arbitration Agency [14]; thus, a larger-scale prospective evaluation of acupuncture safety when performed by KMDs is warranted.

In this context, we conducted a practice-based online survey to establish the safety of acupuncture as currently practised in real-world settings.

## Methods

A prospective study was performed using a previously developed survey form [15] and was approved by the ethics committee of Kyung Hee University (KHSIRB-16-032).

Based on Hanley’s rule of three, we calculated that 30,000 acupuncture treatments were required to identify any AE that occurred more frequently than once per 10,000 treatments [16], which was considered the probability of pneumothorax [17, 18], a rare but serious AE of acupuncture.

From July 2016 to October 2017, KMDs who attained national licensure through state examination after a six-year medical education and were practising at the time of the study were invited by email and text messaging to participate in this study. Participation was not limited by location or type of practice as data were collected via an online system. Those who volunteered to participate provided informed consent. To achieve consistency in data collecting and reporting, we distributed manuals containing instructions on how to fill out survey items to minimise under- or over-reporting and requested participants to complete survey for more than 5 consecutive working days to minimise variances between weekdays and selective reporting. The online survey system was secured so that neither participating KMDs nor study investigators had access to data once submitted.

Information about KMDs, patients' comorbidities and current medications, acupuncture treatment, administrative problems, and local/systemic AEs was collected. Administrative problems included problems due to defective devices and/or incidents that were process-related and deviated from standard practices, e.g., doctor negligence [19]. Because not all administrative problems lead to AEs, KMDs were asked to report whether this occurred. Details regarding data collection [15] are available as online Supplemental Appendix 1.

AEs were analysed in terms of frequency, severity, and causality. Two independent researchers reviewed and classified AE data for severity and causality assessment and any case of disagreement was arbitrated by a panel of drug and intervention safety experts.

Following the Council for International Organisations of Medical Sciences (CIOMS) guidelines [20], we categorised the frequency of administrative problems, local AEs, and systemic AEs as very common (≥1/10), common (≥1/100 and <1/10), uncommon (≥1/1,000 and <1/100), rare (≥1/10,000 and <1/1,000), or very rare (<1/10,000). Frequency data were expressed as number of AEs per 10,000 acupuncture treatments.

AE severity was classified based on the Common Terminology Criteria for Adverse Events (CTCAE) grading scale [21] as mild, moderate, severe, life-threatening, or death (Supplemental Table 1).

AE causality was evaluated using the World Health Organisation-Uppsala Monitoring Centre (WHO-UMC) system for standardised case causality scale [22] as certain, probable/likely, possible, unlikely, conditional/unclassified, or unassessable/unclassifiable (Supplemental Table 2).

All analyses were conducted using SAS 9.4 statistical software (SAS Institute, Cary, NC, USA). Data regarding doctors, patients, acupuncture treatment type, administrative problems, and local/systemic AEs were presented in a descriptive manner. Continuous variables (e.g., patient age) were reported as mean ± standard deviation (SD) and categorical variables were reported as number (percentage).

When analysing associations between AEs and various risk factors, correlation of observations within the same cluster, i.e., individual KMDs was considered using intraclass correlation coefficient (ICC). ICC values between 0.5 and 0.75 were considered indicative of moderate inter-rater reliability and values between 0.75 and 0.9 indicative of good reliability [23]. We implemented a population-averaged model using a generalised estimating equation (GEE) [24, 25] method fitted with the logit link and exchangeable working correlation structure. The GEE model was utilised to test associations between the odds of any kind of local and/or systemic AEs and KMDs’ years of experience, type of practice, and with patients’ gender and age. It was also used to determine relationships between the odds of experiencing AEs due to doctor negligence and KMDs’ years of experience (practice <5 years vs ≥5 years) [26]. Bleeding and bruising, the two most frequently reported AEs in previous prospective studies [3, 4, 27, 28], were also analysed post-hoc using GEE to determine whether they were linked to patient factors such as gender, age, and use of anticoagulants.

An odds ratio (OR) for respective variables was presented with a 95% robust confidence interval (CI).

## Results

In total, 278 KMDs consented to participate in the study; of these, 222 (1.2% of the Association of Korean Medicine members invited to participate) provided data on 37,490 acupuncture treatments (median enrolment period: 129 days; interquartile range: 102-204.5 days). Approximately one-half of participating KMDs (46.8%) had ≥ 5 years of experience; participants practised in private clinics (*n*=103; 46.4%), community health centres (*n*=74; 33.3%), and hospitals (*n*=45; 20.3%). The average age of patients was 59.3 ± 18.5 (mean ± SD) years and 62.5% were female. The most common comorbidity was cardiovascular disease (*n*=11,727; 31.3%) (Table 1).

**Table 1:** Characteristics of Participating KMDs and Patients (*N* = 37,490 acupuncture treatments)

KMDs (*N*=222)	n (%)
Years in practice	
< 5 years	118 (53.2)
≥ 5 years	104 (46.8)
Type of practice	
Hospital	45 (20.3)
Community health centre	74 (33.3)
Private clinic	103 (46.4)
**Patients** (*N*=37,490 treatments)^a^	
Age (years)	59.3 ± 18.5
Gender	
Male	14,058 (37.5)
Female	23,432 (62.5)
Underlying diseases†	
Cardiovascular disease	11,727 (31.3)
Diabetes	4,233 (11.3)
Allergic disease	353 (0.9)
Bleeding disorder	96 (0.3)
Pacemaker	56 (0.1)
Other diseases	5,176 (13.8)
None	21,596 (57.6)
Current medication^b^	
Prescription or over-the-counter drugs	15,841 (42.3)
Antihypertensive drugs	10,685 (28.5)
Anti-diabetic drugs	4,006 (10.7)
Anticoagulant/antiplatelet therapy	2,035 (5.4)
Others including over-the-counter drugs	7,378 (19.7)
Herbal preparations	7,448 (19.9)
Others	865 (2.3)
None	16,241 (43.3)

*KMD* Korean Medicine Doctor

Data are expressed as numbers (percentage) or mean ± SD

^a^Individual patients could be counted more than once if they received multiple treatments

^b^Patients could have ≥1 underlying disease and/or current medication

The most common conditions for which acupuncture was performed in 37,490 treatments were musculoskeletal disorders (*n*=31,971; 85.3%). Manual acupuncture (*n*=35,781; 95.4%), electroacupuncture (*n*=11,110; 29.6%), moxibustion (*n*=8,916; 23.8%), and pharmacopuncture (*n*=3,398; 9.1%) were the most frequently used modalities ( Supplemental Table 3).

A total of 6,645 events (6,199 local or systemic AEs among 4,518 treatments and 446 administrative problems among 434 treatments) were reported. That is, at least one AE was reported in 4,518 out of 37,490 acupuncture treatments, giving a frequency rate of 1,205 per 10,000 acupuncture treatments (95% CI [1,172, 1,238]), classified as “very common.” The four most frequently reported AEs classified as “common” by CIOMS criteria were bleeding, needle site pain, bruising, and redness.

A total of 446 administrative problems due to defective devices or doctor negligence was reported from 434 acupuncture treatments. Of them, the number of acupuncture treatments where only administrative problems without AEs were reported was 250. Of 434 acupuncture treatments with one or more administrative problems, 110 treatments (25.3% of 434 treatments) were reported to have resulted in one or more AEs.

Altogether, the number of acupuncture treatments with at least one AE or administrative problem was 4,768 (4,518 treatments with AEs plus 250 treatments with administrative problems) for a frequency of 1,272 per 10,000 (95% CI [1,238, 1,306]), considered as “very common” (Table 2).

**Table 2:** Frequency of Administrative Problems and AEs Associated with Acupuncture Treatments (*N* = 37,490)

	AE occurrence n (%)^a^	Frequency category by CIOMS^b^
**Administrative problems**		
*Defective devices*		
Defective needle (e.g., broken needle)	31 (0.08)	Rare
*Incidents*		
Delayed needle removal	226 (0.60)	Uncommon
Forgotten or lost needle	136 (0.36)	Uncommon
Burn due to negligence	41 (0.11)	Uncommon
Others	12 (0.03)	Rare
**Local AEs**		
Bleeding	1,969 (5.25)	Common
Needle site pain	1,423 (3.80)	Common
Bruising	951 (2.54)	Common
Paraesthesia	605 (1.61)	Common
Redness	226 (0.60)	Uncommon
Itching	198 (0.53)	Uncommon
Swelling	147 (0.39)	Uncommon
Burn for treatment purpose	38 (0.10)	Uncommon
Blister for treatment purpose	25 (0.07)	Rare
Nerve injury	2 (0.01)	Very rare
Local infection	1 (0.003)	Very rare
Other	17 (0.05)	Rare
**Systemic AEs**		
Tiredness	150 (0.40)	Uncommon
Somnolence	149 (0.40)	Uncommon
Exacerbation of symptoms	79 (0.21)	Uncommon
Lethargy	77 (0.21)	Uncommon
ANS symptoms	45 (0.12)	Uncommon
Pain elsewhere than needling sites	25 (0.07)	Rare
Sleep disturbance	20 (0.05)	Rare
Systemic inflammation	15 (0.04)	Rare
New symptoms after treatment	10 (0.03)	Rare
Convulsion	7 (0.02)	Rare
Emotional complaint	5 (0.01)	Rare
Others	15 (0.04)	Rare

*Abbreviations*: *AE* Adverse event, *ANS* Autonomic nervous system, *CIOMS* Council for International Organisations of Medical Sciences

^a^Denominator was 37,490 acupuncture treatments.

^b^Very common (≥ 1/10), common (≥ 1/100 and < 1/10), uncommon (≥ 1/1,000 and < 1/100), rare (≥ 1/10,000 and < 1/1,000), or very rare (< 1/10,000) [20]

The majority of AEs/administrative problems were mild in severity (*n*=6,497; 97.8% of 6,645). Moderate AEs (n=125, 1.9%) included burn due to negligence (*n*=41), burn for treatment purpose (*n*=38), blister for treatment purpose (*n*=25), systemic inflammation (n=15), nerve injury (*n*=2), fainting as an autonomic nervous system symptom (*n*=1), paresthesia (*n*=1), and other local AEs including mouth dryness after taking herbal medicine containing prepared aconite (*n*=1) and convulsion with chills (*n*=1). Cases of systemic inflammation were not considered serious AEs because they spontaneously disappeared without additional treatment. There were two life-threatening AEs, one of which was anaphylaxis following bee venom pharmacopuncture treatment (the patient was given pheniramine, dexamethasone and epinephrine injection and recovered) and the other was cellulitis in the wrist after manual acupuncture, moxibustion, and cupping treatment (the patient did not take prescribed antibiotics and - following long-distance travel - pain, swelling, and fever worsened, eyesight declined, and patient was hospitalized; following antibiotic treatment, all symptoms resolved and eyesight was normal in 6 weeks) (Supplemental Table 4).

Among 6,645 AEs and administrative problems, 72.9% (*n*=4,843) were determined as certainly associated with acupuncture treatment; these included bleeding, pain at needling site, bruising, burns and blisters for treatment purpose, delayed needle removal, forgotten needles, and/or defective needles. AEs and/or administrative problems assessed as probably/likely or possibly associated with acupuncture accounted for 25.6% (*n*=1,702). It was not possible to determine a causal relationship between acupuncture treatment and some AEs (*n*=94; 1.4%) due to lack of medical information or documentation, e.g., emotional complaints or sleep disturbance following treatment (Supplemental Table 5). Of two life-threatening AEs, anaphylaxis was determined to be possibly associated with bee venom pharmacopuncture treatment and cellulitis was assessed to have a probable association with manual acupuncture needling (Supplemental Table 4).

We examined whether pre-defined factors were associated with AE occurrence in 4,518 acupuncture treatments where at least one local or systemic AE was reported (Table 3). Likelihood of an AE was clustered among KMDs with moderate correlation (ICC: 0.60). KMDs’ clinical experience, type of practice, and patient age did not significantly affect the frequency of overall AE occurrence. The likelihood of local and/or systemic AEs was significantly higher in female patients (adjusted OR: 1.09, 95% CI [1.03-1.16]). KMDs’ clinical experience (<5 years vs ≥5 years) was not associated with incidents (OR: 1.98, 95% CI [0.90-4.35]).

**Table 3:** Factors Associated with AEs, Doctor Negligence, and Bleeding and/or Bruising (*N*=37,490 acupuncture treatments)

Variables	Unadjusted OR (95% CI)	Robust SE	*P*-value	Adjusted OR (95% CI)	Robust SE	*P*-value
**AEs**						
*KMDs*						
Years in practice						
< 5 years	1.00			1.00		
≥ 5 years	1.42 (0.94-2.14)	0.209	0.095	1.35 (0.86-2.12)	0.229	0.189
Type of practice						
Hospital	1.00			1.00		
Private clinic	1.54 (0.84-2.84)	0.312	0.165	1.51 (0.83-2.77)	0.308	0.178
Community health centre	1.22 (0.65-2.30)	0.323	0.534	1.37 (0.73-2.59)	0.325	0.330
*Patients*						
Age^a^	1.00 (1.00-1.00)	0.001	0.297	1.00 (1.00-1.00)	0.001	0.219
Gender						
Male	1.00			1.00		
Female	1.09 (1.02-1.16)	0.032	0.009	1.09 (1.03-1.16)	0.031	0.005
**Incidents**						
*Years in practice*						
< 5 years	1.98 (0.90-4.35)	0.402	0.089			
≥ 5 years	1.00					
**Bleeding and/or bruising**						
*Age*^b^						
< 40 years	1.00			1.00		
≥ 40 years	1.15 (1.02-1.22)	0.068	0.044	1.13 (0.98-1.29)	0.070	0.084
*Gender*						
Male	1.00			1.00		
Female	1.12 (1.02-1.22)	0.044	0.013	1.12 (1.02-1.22)	0.045	0.014
*Oral anticoagulant*	1.27 (1.07-1.50)	0.087	0.068	1.26 (1.06-1.50)	0.088	0.009

*Abbreviations*: *OR* Odds ratio; *CI* Confidence interval, *SE* Standard error, *AE* Adverse event, *KMD* Korean Medicine Doctor

^a^As age was modeled as a continuous variable in testing association with AEs; OR of age meant change by one year increase

^b^Age was coded as a dichotomous variable since oral anticoagulant is an age-dependent treatment

Female patients were more likely to experience bleeding/bruising compared with males. The adjusted model showed that female gender (adjusted OR: 1.12, 95% CI [1.02-1.22]) and use of anticoagulants (adjusted OR: 1.26, 95% CI [1.06-1.50]) had a significant impact on the likelihood of experiencing bleeding and/or bruising and a significant interaction between gender and use of anticoagulants was observed (Table 3).

## DISCUSSION

In this large-scale, prospective practice-based survey study in which 222 KMDs participated, at least one AE was reported in 4,518 out of a total of 37,490 acupuncture treatments (frequency rate: 1,205 per 10,000). The frequency increases to 1,272 per 10,000 (4,768 out of 37,490) when administrative problems were added, regardless of whether patient harm occurred. Although AEs occurred very commonly, most were mild or moderate in severity. Common AEs included bleeding, needle site pain, and bruising, all of which were classified as certainly associated with acupuncture treatment. Two life-threatening AEs were reported and both patients recovered following treatment with no negative sequelae.

The frequency of AEs and/or administrative problems in the current study is substantially higher compared with previous reports: 14/10,000 treatments in Japan [5]; 111/10,000 treatments in Germany [4]; 320/10,000 treatments in Korea [11]; and 684/10,000 consultations in the UK among medical doctors and physiotherapists [3, 26]. One exception was the UK acupuncturist survey, which reported 1,316 AEs/10,000 acupuncture treatments [29]. This discrepancy may be due partly to differences in: provider training, clinical expertise, and years in practice; patient age/gender, bleeding tendency, and underlying diseases; acupuncture technique such as trigger point/dry needling/Toyohari, or inclusion of bee venom pharmacopuncture or moxibustion; inclusion of problems caused by defective needles or doctors’ negligence; and data collection methods such as intensive monitoring or online survey.

Commonly reported AEs in the present study included bleeding, needle site pain, and bruising, which are similar to previous prospective studies [3, 4, 8, 11, 28, 29]. However, direct comparison may be inappropriate because safety profiles varied greatly in terms of applied acupuncture modalities, types of participating patients and practitioners, and definition of AEs and/or administrative problems [4, 30, 31]. An international consensus of how and what AEs are measured and how they should be reported would make it easier to directly compare acupuncture safety studies across countries.

Although most AEs and administrative problems in this study were mild to moderate in severity, rare but serious AEs should not be ignored. One life-threatening AE was anaphylactic shock after bee venom pharmacopuncture treatment. Judging from previous studies [8, 32] documenting an association between use of bee venom and AEs, it is critically important for KMDs to adhere to current first aid training as in the curriculum of colleges of Korean Medicine to insure patient safety.

A second life-threatening AE was cellulitis with uveitis after acupuncture treatment following a traffic accident. Although local infection and/or systemic inflammation were very rare or rare and were typically mild (and the link between acupuncture needling and uveitis was not clear), it can be prevented almost entirely by enhancing preexisting hygiene training and clean needle technique education, not only in the regular curriculum of Korean Medicine but also in continuing education programmes.

Pneumothorax has been reported in previous studies as a rare but serious acupuncture-associated AE [18, 30, 31, 33, 34], but was not observed in this study. A recent study [8] of safety of acupuncture treatment by KMDs also reported that pneumothorax was very rare (incidence rate 0.005%) and symptoms were transient. While the possibility of underreporting serious AEs cannot be completely excluded, absence of pneumothorax in the present study may be partly attributed to improved training in anatomy and meridian/acupoints in colleges of Korean Medicine in recent years [35, 36].

When KMDs’ clinical experience/type of practice and patients’ age were adjusted, female patients were found to be significantly more likely to experience AEs (OR: 1.09, 95% CI [1.03-1.16]) compared with males, similar to previous findings [37]. Bleeding/bruising accounted for more than half of all AEs, and female patients were at considerably higher risk for these events compared with male patients, which may explain their higher odds. With regard to KMDs’ competence, years of practice, or clinical experience, these factors did not appear related to AE occurrence. Administrative problems due to doctor negligence also appeared unlikely to produce significantly more AEs, consistent with the UK study in which no association between AE frequency and acupuncture training or clinical experience was found [26].

To our knowledge this is the first and largest practice-based study on acupuncture safety in Korea. Survey questions were simple and intuitive, answered by ticking checkboxes online. A total of 222 KMDs voluntarily participated in the survey, resulting in a substantial volume of data on 37,490 treatments [15]. Data were collected without any modification of usual acupuncture practice, such that results reflect current real-world Korean Medicine practice to ensure the validity of study findings. KMD participants represent different practice types and reported on modalities that included not only manual acupuncture, but also techniques such as pharmacopuncture, bee venom pharmacopuncture, warm needling, and cupping, some of which may be infrequently used in other countries. In addition, we collected data related to administrative problems due to defective devices or negligence to comprehensively assess safety issues.

Nevertheless, our study has some limitations. Despite efforts to recruit participants, only 222 of 18,654 KMDs (response rate 1.2%) voluntarily participated in the study. Given that 300-600 KMDs usually respond to a one-time survey and the present study required participants to input data over a period of five consecutive days [38, 39], the enrolment rate is understandable but nonetheless may be considered low.

Another limitation is the possibility of non-response bias. Due to the web-based survey design, KMDs with undependable internet access or those with limited computer skills may have been excluded; further, doctors working in health community centres (33.3% in this study) may represent a younger cohort as they are typically engaged in post-graduate military service compared with the nationwide proportion (4.9%) [36], which could affect study results. However, practice type did not seem to significantly impact AE frequency (Table 3).

This study could be prone to participant reporting bias [40]. While findings are similar to those of previous studies [3, 4, 8, 11, 28, 29], underreporting of serious AEs and/or overreporting of minor/transient AEs such as bleeding and needle site pain may occur. Future surveys should consider having an independent observer report and assess the AEs.

Some may argue that this study is not entirely free from non-negligible availability bias [41] because a majority of reported acupuncture modalities were manual acupuncture (95.4%). However, according to Korean Medicine statistical data [36], manual acupuncture is the most commonly used method, and our results do reflect everyday acupuncture practice.

Lastly, because severity and causality were analysed by independent researchers and experts based on information provided by KMDs, assessment results may not be consistent with participating KMDs’ evaluations. In the future, it would be informative to investigate whether or not practitioners reporting AEs and independent researchers/experts’ assessments of severity and causality may disagree.

It is important to share our findings with KMDs to help them reflect on their current acupuncture practices and to modify them if necessary to improve patient safety. Informing them of actions that might be taken to reduce potentially avoidable adverse incidents such as burns and blisters for treatment purpose, delayed needle removal, or forgotten needles, can be helpful.

Of note, some KMDs reported AEs more often than others, producing a “clustering” effect. Given that this phenomenon was reported in previous studies [11, 37], further research of provider characteristics beyond clinical experience or duration of acupuncture training (that were not associated with increased AE reporting in this study) is required to ascertain what, if any, other factors such as types of acupuncture modalities/styles or stimulation/manipulation methods might contribute.

If issues of overreporting minor AEs and underreporting serious AEs occur based solely on data collected from doctors, as in the present study, the addition of qualitative investigation of patients’ experiences and perspectives on acupuncture-related AEs could help to balance this potential limitation [37].

Previous studies of acupuncture safety used various definitions and criteria for AEs, making direct comparison difficult; for instance, some studies included incidents due to doctor’s negligence [4, 29] whereas others did not [42], and the definition of bleeding varied [15, 28, 31]. Development and implementation of a standardised data collection form could facilitate comparison of AEs across countries or different acupuncture styles/techniques. As frequency, type, and consequences of AEs may vary among different patient subpopulations, e.g., pregnant women, further studies of acupuncture-associated AEs by subgroups are justifiable.

## Conclusions

Although frequency of overall AEs associated with acupuncture treatment performed by KMDs were very common, they were transient and mostly mild. Acupuncture treatment provided by qualified KMDs may be considered a safe treatment.

## Supplementary Information

**Additional file 1: Appendix 1.** Collecting items in an online questionnaire. **Supplemental Table 1.** Grading scale of severity of AEs. **Supplemental Table 2.b **WHO-UMC Causality categories. **Supplemental Table 3.** Information associated with acupuncture treatment (*N* = 37,490 acupuncture treatments). **Supplemental Table 4.** Serious adverse event reports. **Supplemental Table 5.** Causality assessment using WHO-UMC causality categories (*N* = 37,490 acupuncture treatments).

## Data Availability

The datasets used and/or analysed during the current study are available from the corresponding author on reasonable request.

## References

[1] Linde K, Vickers A, Hondras M, ter Riet G, Thormählen J, Berman B (2001). Systematic reviews of complementary therapies - an annotated bibliography. part 1: acupuncture. BMC Complement Altern Med.

[2] Vickers AJ, Vertosick EA, Lewith G, MacPherson H, Foster NE, Sherman KJ (2018). Acupuncture for chronic pain: update of an individual patient data meta-analysis. J Pain.

[3] White A, Hayhoe S, Hart A, Ernst E (2001). Adverse events following acupuncture: prospective survey of 32 000 consultations with doctors and physiotherapists. BMJ.

[4] Witt CM, Pach D, Brinkhaus B, Wruck K, Tag B, Mank S (2009). Safety of acupuncture: results of a prospective observational study with 229,230 patients and introduction of a medical information and consent form. Forsch Komplementärmed.

[5] Yamashita H, Tsukayama H, Tanno Y, Nishijo K (1999). Adverse events in acupuncture and moxibustion treatment: a six-year survey at a national clinic in Japan. J Altern Complement Med.

[6] Kim J, Kang DI (2010). A descriptive statistical approach to the Korean pharmacopuncture therapy. J Acupunct Meridian Stud.

[7] World Health Organization. Acupuncture: Review and Analysis of Reports on Controlled Clinical Trials. Geneva: World Health Organization; 2002.

[8] Kim MR, Shin JS, Lee J, Lee YJ, Ahn YJ, Park KB (2016). Safety of acupuncture and pharmacopuncture in 80,523 musculoskeletal disorder patients: a retrospective review of internal safety inspection and electronic medical records. Medicine.

[9] Park SU, Ko CN, Bae HS, Jung WS, Moon SK, Cho KH (2009). Short-term reactions to acupuncture treatment and adverse events following acupuncture: a cross-sectional survey of patient reports in Korea. J Altern Complement Med.

[10] Kim SM, Kim Y. 2020 National Health Insurance Statistical Annual Report: Health Insurance Review & Assessment Service and National Health Insurance Service: Wonju; 2021.

[11] Park JE, Lee MS, Choi JY, Kim BY, Choi SM (2010). Adverse events associated with acupuncture: a prospective survey. J Altern Complement Med.

[12] Bensoussan A, Myers SP, Carlton AL (2000). Risks associated with the practice of traditional Chinese medicine: an Australian study. Arch Fam Med.

[13] Lee HJ, Kim YJ, Kim WY (2017). Safety concerns with thoracoabdominal acupuncture: Experience at a tertiary-care emergency department.. Pain Med.

[14] Hwang HW, Lee BH, Kim KH (2017). Claimed adverse events of Korean Medicine in South Korea: analysis of cases in the Korea Medical Dispute Mediation and Arbitration Agency databases. Korean J Acupuncture.

[15] Kim SY, Lee JH, Yook TH, Park JM, Leem JT, Lee HS (2015). Development and validation of a survey form for adverse events associated with acupuncture and moxibustion. Korean J Acupuncture.

[16] Eypasch E, Lefering R, Kum CK, Troidl H (1995). Probability of adverse events that have not yet occurred: a statistical reminder. BMJ.

[17] White A. A cumulative review of the range and incidence of significant adverse events associated with acupuncture. Acupunct Med. 2004;22:122–33. 10.1136/aim.22.3.122 15551936

[18] Ernst E, White A (1997). Life-threatening adverse reactions after acupuncture? A systematic review. Pain.

[19] World Health Organisation. World alliance for patient safety: WHO draft guidelines for adverse event reporting and learning systems from information to action. Geneva: World Health Organisation; 2005. https://apps.who.int/iris/handle/10665/69797.

[20] European Commission. A guideline on summary of product characteristics (SmPC). 2009. https://health.ec.europa.eu/system/files/2016-11/smpc_guideline_rev2_en_0.pdf.

[21] US Department of Health and Human Services. Common terminology criteria for adverse events (CTCAE) version 4.0. Bethesda: National Institutes of Health, National Cancer Institute; 2009.

[22] World Health Organisation Uppsala Monitoring Center. The use of the WHO-UMC system for standardised case causality assessment. 2018. https://who-umc.org/media/164200/who-umc-causality-assessment_new-logo.pdf.

[23] Koo TK, Li MY (2016). A guideline of selecting and reporting intraclass correlation coefficients for reliability research. J Chiropr Med.

[24] Hubbard AE, Ahern J, Fleischer NL, Van der Laan M, Lippman SA, Jewell N (2010). To GEE or not to GEE: comparing population average and mixed models for estimating the associations between neighborhood risk factors and health. Epidemiology.

[25] Zeger SL, Liang KY, Albert PS (1988). Models for longitudinal data: a generalized estimating equation approach. Biometric.

[26] White A, Hayhoe S, Hart A, Ernst E (2001). Survey of adverse events following acupuncture (SAFA): a prospective study of 32,000 consultations. Acupunct Med.

[27] Furuse N, Shinbara H, Uehara A, Sugawara M, Yamazaki T, Hosaka M (2017). A multicenter prospective survey of adverse events associated with acupuncture and moxibustion in Japan. Med Acupunct.

[28] Yamashita H, Tsukayama H, Hori N, Kimura T, Tanno Y (2000). Incidence of adverse reactions associated with acupuncture. J Altern Complement Med.

[29] MacPherson H, Thomas K, Walters S, Fitter M (2001). The York acupuncture safety study: prospective survey of 34 000 treatments by traditional acupuncturists. BMJ.

[30] Ernst E, White AR (2001). Prospective studies of the safety of acupuncture: a systematic review. Am J Med..

[31] Bäumler P, Zhang W, Stübinger T, Irnich D (2021). Acupuncture-related adverse events: systematic review and meta-analyses of prospective clinical studies. BMJ Open.

[32] Park JH, Yim BK, Lee JH, Lee S, Kim TH (2015). Risk associated with bee venom therapy: a systematic review and meta-analysis. PLoS One.

[33] Kim YJ, Kim SH, Lee HJ, Kim WY (2018). Infectious adverse events following acupuncture: clinical progress and microbiological etiology. J Korean Med Sci.

[34] Ernst E, Lee MS, Choi TY (2011). Acupuncture: does it alleviate pain and are there serious risks? A review of reviews. Pain.

[35] Baek KH, Park YL (2015). A legal study on feasibility to instruct physical therapists by Korean Medicine Doctors. Law Policy.

[36] Publication Committee for Yearbook of Traditional Korean Medicine. 2016 Yearbook of Traditional Korean Medicine: Korean Institute of Oriental Medicine: Yuseong; 2018.

[37] MacPherson H, Scullion A, Thomas KJ, Walters S (2004). Patient reports of adverse events associated with acupuncture treatment: a prospective national survey. Qual Saf Health Care.

[38] Woo HL, Ji HR, Park KS, Hwang DS, Lee CH, Jang JB (2017). A survey on Korean Medicine Doctors’ recognition and clinical fields of treating primary dysmenorrhea for developing Korean medicine clinical practice guideline for dysmenorrhea. J Korean Obstet Gynecol.

[39] Yeo MK, Park KH, Lee YS (2017). A national-wide survey on utilization of pattern identification for chronic diseases among Korean Medicine Doctors. J Soc Prev Korean Med.

[40] Dwan K, Altman DG, Arnaiz JA, Bloom J, Chan AW, Cronin E (2008). Systematic review of the empirical evidence of study publication bias and outcome reporting bias. PLoS One.

[41] Mamede S, van Gog T, van den Berge K, Rikers RM, van Saase JL, van Guldener C (2010). Effect of availability bias and reflective reasoning on diagnostic accuracy among internal medicine residents. JAMA.

[42] Ernst G, Strzyz H, Hagmeister H (2003). Incidence of adverse effects during acupuncture therapy-a multicentre survey. Complement Ther Med.

