# Supplementary material for: Safety of acupuncture by Korean Medicine Doctors: a prospective, practice-based survey of 37,490 consultations
Source: BMC Complement Med Ther. 2022 Nov 18;22:300. doi: 10.1186/s12906-022-03782-z (PMC9675262; doi:10.1186/s12906-022-03782-z)
Supplement: Supplementary file 1 — Additional file 1: Appendix 1. Collecting items in an online questionnaire. Supplemental Table 1. Grading scale of severity of AEs. Supplemental Table 2.b WHO-UMC Causality categories. Supplemental Table 3. Information associated with acupuncture treatment (N = 37,490 acupuncture treatments). Supplemental Table 4. Serious adverse event reports. Supplemental Table 5. Causality assessment using WHO-UMC causality categories (N = 37,490 acupuncture treatments). [file 12906_2022_3782_MOESM1_ESM.docx]

**Supplemental Material**

**Appendix 1.** Collecting items in an online questionnaire

Following information was collected in a questionnaire which consists of five parts: Korean Medicine Doctors, patients, acupuncture treatment, administrative problems, local and systemic adverse events (AEs). Participating Korean Medicine Doctors were allowed to select multiple choices regarding patient's underlying diseases, current medication, conditions for acupuncture treatment, and details of acupuncture treatment.

[Korean Medicine Doctors]

- Years in practice

□_1_ < 5 years □_2_ ≥ 5 years

- Type of practice

□_1_ Hospital □_2_ Private clinic □_3_ Community health centre □_4_ Others

[Patients]

- Gender

□_1_ Male □_2_ Female (Pregnancy: □_1_ Yes □_2_ No)

- Age
- Current medication (multiple choices allowed)

□_1_ Prescription or over-the-counter drugs (□_1_ Anticoagulants/antiplatelet therapy □_2_ Antihypertensive drugs □_3_ Anti-diabetic drugs □_4_ Others including over-the-counter drugs) □_2_ Herbal preparations □_3_ Others

- Underlying diseases (multiple choices allowed)

□_1_ Cardiovascular disease □_2_ Diabetes □_3_ Bleeding disorder □_4_ Allergic disease □_5_ Pacemaker □_6_ Other diseases

[Acupuncture treatment]

- Conditions for acupuncture treatment (multiple choices allowed)

□_1_ Infectious disease □_2_ Endocrine system □_3_ Nervous system □_4_ Ear-nose-and-throat (ENT) □_5_ Respiratory system □_6_ Dermatology

□_7_ Genitourinary system □_8_ Oncology □_9_ Mental/ behavioral disorders □_10_ Ophthalmology □_11_ Circulatory system □_12_ Digestive system

□_13_ Musculoskeletal system □_14_ Obstetrics and gynaecology (OBGY) □_15_ Paediatrics □_16_ Others

- Type of acupuncture treatment (multiple choices allowed)

□_1_ Manual acupuncture □_2_ Electroacupuncture □_3_ Intradermal acupuncture/ ear acupuncture □_4_ Warm needling □_5_ Fire needling

□_6_ Moxibustion □_7_ Bee venom pharmacopuncture □_8_ Pharmacopuncture □_9_ Others

- Treatment parts in the body (multiple choices allowed)

□_1_ Head □_2_ Face □_3_ Shoulder □_4_ Low back □_5_ Flank □_6_ Hip □_7_ Thorax □_8_ Abdomen □_9_ Upper limbs □_10_ Lower limbs □_11_ Others

[Administrative problems]

- Type of administrative problems (multiple choices allowed)

□_1_ Delayed needle removal □_2_ Forgotten or lost needle □_3_ Defective devices (e.g., broken needles) □_4_ Burn due to negligence □_4_ Others

[AEs]

- Type of AEs (multiple choices allowed)
- Local AEs

□_1_ Needling site pain □_2_ Bleeding □_3_ Bruising □_4_ Swelling □_5_ Itching □_6_ Redness □_7_ Paraesthesia □_8_ Burn for treatment purpose □_9_ Local infection □_10_ Blister for treatment purpose □_11_ Nerve injury □_12_ Organ injury □_13_ Pneumothorax □_14_ Others

- Systemic AEs

□_1_ Exacerbations of symptoms □_2_ Tiredness □_3_ Lethargy □_4_ Somnolence

□_5_ Autonomic nervous system (ANS) symptoms

□_1_ Nausea □_2_ Vomiting □_3_ Headache □_4_ Dizziness □_5_ Sweating □_6_ Faint □_7_ Syncope □_8_ Dyspnea □_9_ Palpitation □_10_ Tachycardia □_11_ Constipation □_12_ Diarrhea □_13_ Others

□_6_ Pain elsewhere than needling sites □_7_ New symptoms after treatment □_8_ Sleep disturbance

□_9_ Emotional complaint

□_1_ Anxiety □_2_ Nervousness □_3_ Fear □_4_ Others

□_10_ Convulsion □_11_ Systemic inflammation □_12_ Others

**Supplemental table 1.** Grading scale of severity of AEs

| Grade | | Severity of the AEs | Clinical descriptions |
| --- | --- | --- | --- |
|  | 1 | Mild | - Mild - Asymptomatic or mild symptom - Clinical or diagnostic observations only - Intervention not indicated |
|  | 2 | Moderate | - Moderate - Minimal, local or noninvasive intervention indicated - Limiting age-appropriate instrumental ADL* |
|  | 3 | Severe | - Severe or medically significant but not immediately life-threatening - Hospitalisation or prolongation of hospitalisation indicated - Disabling; limiting self care ADL† |
|  | 4 | Life-threatening | - Life-threatening consequences - Urgent intervention indicated |
|  | 5 | Death | - Death related to AE |

ADL, Activities of Daily Living; AE, adverse event.

*, Instrumental ADL refers to preparing meals, shopping for groceries or clothes, using the telephone, managing money, etc.

†, Self care ADL refers to bathing, dressing and undressing, feeding self, using the toilet, taking medications, and not bedridden.

**Supplemental table 2.** WHO-UMC Causality categories

| Causality term | | Assessment criteria* |
| --- | --- | --- |
|  | Certain | - Event or laboratory test abnormality, with plausible time relationship to drug intake - Cannot be explained by disease or other drugs - Response to withdrawal plausible (pharmacologically, pathologically) - Event definitive pharmacologically or phenomenologically (i.e. an objective and specific medical disorder or a recognised pharmacological phenomenon) - Rechallenge satisfactory, if necessary |
|  | Probable/  Likely | - Event or laboratory test abnormality, with reasonable time relationship to drug intake - Unlikely to be attributed to disease or other drugs - Response to withdrawal clinically reasonable - Rechallenge not required |
|  | Possible | - Event or laboratory test abnormality, with reasonable time relationship to drug intake - Could also be explained by disease or other drugs - Information on drug withdrawal may be lacking or unclear |
|  | Unlikely | - Event or laboratory test abnormality, with a time to drug intake that makes a relationship improbable (but not impossible) - Disease or other drugs provide plausible explanations |
|  | Conditional/  Unclassified | - Event or laboratory test abnormality - More data for proper assessment needed, or - Additional data under examination |
|  | Unassessable/  Unclassifiable | - Report suggesting an adverse reaction - Cannot be judged because information is insufficient or contradictory - Data cannot be supplemented or verified |

WHO-UMC, World Health Organisation-Uppsala Monitoring Centre.

*, All points should be reasonably complied with.

**Supplemental table 3.** Information associated with acupuncture treatment (N = 37,490 acupuncture treatments)

| Diseases/conditions for acupuncture treatment* | | | n (%)† |
| --- | --- | --- | --- |
|  | Musculoskeletal system | | 31,971 (85.3) |
|  | Nervous system | | 4,971 (13.3) |
|  | Digestive system | | 2,825 (7.5) |
|  | Circulatory system | | 1,163 (3.1) |
|  | ENT | | 777 (2.1) |
|  | OBGY | | 711 (1.9) |
|  | Mental/behavioral disorder | | 589 (1.6) |
|  | Genitourinary system | | 546 (1.5) |
|  | Respiratory system | | 470 (1.3) |
|  | Endocrine system | | 432 (1.2) |
|  | Paediatrics | | 280 (0.7) |
|  | Dermatology | | 257 (0.7) |
|  | Ophthalmology | | 97 (0.3) |
|  | Oncology | | 63 (0.2) |
|  | Infectious disease | | 23 (0.1) |
|  | Others | | 656 (1.7) |
| Acupuncture Treatment* | | |  |
|  | *Type of acupuncture treatment* | |  |
|  |  | Manual acupuncture | 35,781 (95.4) |
|  |  | Electroacupuncture | 11,110 (29.6) |
|  |  | Moxibustion | 8,916 (23.8) |
|  |  | Pharmacopuncture | 3,398 (9.1) |
|  |  | Cupping | 3,178 (8.5) |
|  |  | Bee venom pharmacopuncture | 1,056 (2.8) |
|  |  | Warm needling | 682 (1.8) |
|  |  | Intradermal needling including ear acupuncture | 485 (1.3) |
|  |  | Fire needling | 98 (0.3) |
|  |  | Others | 1,208 (3.2) |
|  | *Treatment parts in the body* | |  |
|  |  | Lower limbs | 19.896 (53.1) |
|  |  | Upper limbs | 15,940 (42.5) |
|  |  | Low back | 13,093 (34.9) |
|  |  | Shoulder | 9,664 (25.8) |
|  |  | Head | 9,301 (24.8) |
|  |  | Abdomen | 4,170 (11.1) |
|  |  | Hip | 2,837 (7.6) |
|  |  | Face | 2,407 (6.4) |
|  |  | Flank | 356 (0.9) |
|  |  | Thorax | 308 (0.8) |
|  |  | Others | 92 (0.2) |

ENT, Ear-nose-and-throat; OBGY, Obstetrics and gynaecology.

*, Korean Medicine Doctors were allowed to select multiple choices regarding diseases/conditions for acupuncture treatment and details of acupuncture treatment modalities.

†, percentage was calculated by dividing a corresponding number by 37,490 acupuncture treatments.

**Supplemental table 4.** Serious adverse event reports

| Serious AEs | Details and follow-ups |
| --- | --- |
| Anaphylaxis | - A 58-year-old female patient with chronic low back pain - She was given bee venom pharmacopuncture treatment at bilateral BL23 in the lower back and within 30 minutes, developed redness and itching at the treated parts at her lower back which then spreaded to her chest and all over her body. - Pheniramine (one ampoule, 4 mg, i.m.) injection and ice pack was applied but symptoms got worse. Another pheniramine ampoule (i.v.) and dexamethasone injection (i.m.) was then given. - Blood pressure rapidly dropped to 90/50 mmHg, and she felt lightheaded, falling to the floor. Blood pressure was then 70/30 mmHg, body temperature 35.8°C, and pulse 89 beats/min. - Epinephrine injection was given three times (total 0.3 mL, i.m.); the above symptoms were relieved and she recovered completely in five hours. |
| Cellulitis/  uveitis | - A 27-year-old female patient with shoulder and wrist pain after traffic accident - She was given acupuncture treatment for her shoulder and wrist pain. - Ten days later, she visited again and received manual acupuncture, moxibustion, and cupping treatment. - The next day, the wrist pain became worse and developed redness and swelling in her wrist. - Three days later, she complained temperature and chill, visited western medical hospital, was diagnosed as cellulitis and antibiotics were prescribed. But she traveled a long distance to attend relative’s wedding without taking antibiotics, and felt even more stressed and tired. - She was then hospitalised for exacerbating pain, and fever and chill; had a decline in her eye sight, and was additionally diagnosed as uveitis. - She took antibiotics and all symptoms were eliminated in six weeks, with eye sight back to normal. |

AE, adverse event; BL23, 23^rd^ acupoint in the urinary bladder meridian by WHO standard acupoint nomenclature; i.m., intramuscular; i.v., intravenous.

**Supplemental table 5.** Causality assessment using WHO-UMC causality categories (N = 37,490 acupuncture treatments)

| Causality | n | AEs | n of AEs (%)† |
| --- | --- | --- | --- |
| Certain | 4,843 | Bleeding | 1,969 (5.3) |
|  |  | Needling site pain | 1,423 (3.8) |
|  |  | Bruising | 951 (2.5) |
|  |  | Delayed needle removal | 226 (0.6) |
|  |  | Forgotten or lost needle | 136 (0.4) |
|  |  | Burn for treatment purpose | 38 (0.1) |
|  |  | Burn due to negligence | 34 (0.09) |
|  |  | Defective needle | 31 (0.08) |
|  |  | Blisters for treatment purpose | 25 (0.06) |
|  |  | Other administrative problems | 10 (0.03) |
| Probable/Likely | 6 | Autonomic nervous system symptoms‡ | 4 (0.01) |
|  |  | Local infection | 1 (0.003) |
|  |  | Nerve injury | 1 (0.003) |
| Possible | 1,702 | Paraesthesia | 604 (1.6) |
|  |  | Redness | 226 (0.6) |
|  |  | Itching | 198 (0.5) |
|  |  | Tiredness | 150 (0.4) |
|  |  | Somnolence | 149 (0.4) |
|  |  | Swelling | 147 (0.4) |
|  |  | Exacerbation of symptoms | 79 (0.2) |
|  |  | Lethargy | 77 (0.2) |
|  |  | Autonomic nervous system symptoms | 26 (0.07) |
|  |  | Sleep disturbance | 20 (0.05) |
|  |  | Other local AEs | 13 (0.03) |
|  |  | Other systemic AEs | 12 (0.03) |
|  |  | Convulsion | 1 (0.003) |
| Unlikely | - |  |  |
| Conditional/Unclassified | - |  |  |
| Unassessable/Unclassifiable§ | 94 | Pain elsewhere than needling sites | 25 (0.07) |
|  |  | Autonomic nervous system symptoms | 15 (0.04) |
|  |  | Systemic inflammation | 15 (0.04) |
|  |  | New symptoms after treatment | 10 (0.03) |
|  |  | Burn due to negligence | 7 (0.02) |
|  |  | Convulsion | 6 (0.02) |
|  |  | Emotional complaint | 5 (0.01) |
|  |  | Other local AEs | 4 (0.01) |
|  |  | Other systemic AEs | 3 (0.008) |
|  |  | Other administrative problem | 2 (0.005) |
|  |  | Nerve injury | 1 (0.003) |
|  |  | Paraethesia | 1 (0.003) |
| Total | 6,645 |  |  |

WHO-UMC, World Health Organisation-Uppsala Monitoring Centre; AE, adverse event.

†, percentage was calculated by dividing a corresponding number by 37,490, i.e. a total number of acupuncture treatments.

‡, autonomic nervous system symptoms included faint (n=3) and syncope (n=1).

§, Ninety-five AEs and administrative problems were considered unassessable/unclassifiable due to a lack of information regarding type of acupuncture treatment, treatment parts in the body, and/or other details necessary for categorisaion.
